# Expression of the central growth regulator *BIG BROTHER *is regulated by multiple *cis*-elements

**DOI:** 10.1186/1471-2229-12-41

**Published:** 2012-03-20

**Authors:** Holger Breuninger, Michael Lenhard

**Affiliations:** 1 Institut für Biochemie und Biologie, Universität Potsdam, Karl-LiebknechtStr.24-25, D-14476 Potsdam, Germany; 2 Department of Plant Sciences, University of Oxford, South Parks Road, Oxford OX1 3RB, UK

## Abstract

**Background:**

Much of the organismal variation we observe in nature is due to differences in organ size. The observation that even closely related species can show large, stably inherited differences in organ size indicates a strong genetic component to the control of organ size. Despite recent progress in identifying factors controlling organ growth in plants, our overall understanding of this process remains limited, partly because the individual factors have not yet been connected into larger regulatory pathways or networks. To begin addressing this aim, we have studied the upstream regulation of expression of *BIG BROTHER *(*BB*), a central growth-control gene in *Arabidopsis thaliana *that prevents overgrowth of organs. Final organ size and *BB *expression levels are tightly correlated, implying the need for precise control of its expression. *BB *expression mirrors proliferative activity, yet the gene functions to limit proliferation, suggesting that it acts in an incoherent feedforward loop downstream of growth activators to prevent over-proliferation.

**Results:**

To investigate the upstream regulation of *BB *we combined a promoter deletion analysis with a phylogenetic footprinting approach. We were able to narrow down important, highly conserved, *cis*-regulatory elements within the *BB *promoter. Promoter sequences of other Brassicaceae species were able to partially complement the *A. thaliana bb-1 *mutant, suggesting that at least within the Brassicaceae family the regulatory pathways are conserved.

**Conclusions:**

This work underlines the complexity involved in precise quantitative control of gene expression and lays the foundation for identifying important upstream regulators that determine *BB *expression levels and thus final organ size.

## Background

The control of plant organ size and therefore biomass is a complex trait. Plants of a given species will grow to a characteristic size, indicating an intrinsic and hence genetic control for organ size. Plant organs grow in two phases: Initially the primordium grows mainly by cell proliferation. This phase is followed by a cell expansion phase where the organ mainly grows by taking up water and increasing the cell size. In recent years interest in the genetic basis of plant organ size has grown and our understanding of pathways contributing to this trait has greatly improved (see [1]). However, our global understanding of the control of organ size is still limited. Previous research was able to identify a large number of components and pathways all contributing to the final size of an organ by either controlling cell number, cell size or both, but we still miss a clear understanding of how these different pathways interact and how their activities are integrated. One way to address this issue is to follow up on the regulation of central components of organ size control (for review see [1-4]).

The *BIG BROTHER *(*BB*) gene, encoding an E3 ubiquitin ligase, represents a central regulator of organ size. Plants lacking *BB *activity form larger organs. Conversely, plants expressing higher levels of *BB *produce smaller organs, indicating that *BB *acts as a negative regulator for organ size. These effects on size were strictly dosage dependent, i.e. the lower dosage of *BB *in *bb-1/+ *heterozygous plants led to half the increase in organ size compared to *bb-1 *homozygous plants. Analysis of the growth dynamics in *bb-1 *mutants showed that *BB *restricts the cell proliferation phase. Cells of *bb-1 *mutants divide for a longer period of time and start elongating later, indicating that *BB *activity is required to limit excessive cell proliferation. *BB *expression was detected in all proliferating tissues, suggesting a plant-wide function in limiting cell divisions. In growing organs levels of *BB *expression closely mirrored mitotic activity; the highest levels of expression were detected in the early stages of organ growth with high mitotic activity, and *BB *expression appeared to decline in concert with the decrease in cell divisions. This somewhat counterintuitive expression pattern suggests that *BB *functions as an intrinsic growth brake in an incoherent feedforward loop [5] during organ growth. According to this idea, *BB *expression would be induced by (a) factor(s) that also activate(s) cell proliferation, yet it in turn would counteract this growth stimulation. Such a system could be used to fine-tune proliferation, preventing over-proliferation, while still allowing for the appropriate number of cells to be formed [6].

Testing this idea and also understanding how the level of *BB *activity is controlled so as to ensure the formation of appropriately sized organs will ultimately require the identification of upstream regulators that promote or inhibit *BB *expression. As a first step towards this goal, we analyzed the *BB *promoter for important *cis*-regulatory elements. By combining a promoter deletion approach with phylogenetic footprinting [7-9] we show that *BB *expression is regulated by a combination of different *cis*-elements that show complex interactions. We identify four highly conserved promoter elements, each of which is dispensable for *BB *expression; however, the combined loss of some or all of these elements strongly interferes with *BB *promoter activity. These studies provide a baseline for understanding the upstream regulation of this central growth control gene.

## Results and discussion

### The *BB *promoter contains *cis*-elements in the 5' UTR and 5' non-transcribed region

**Figure 1:**
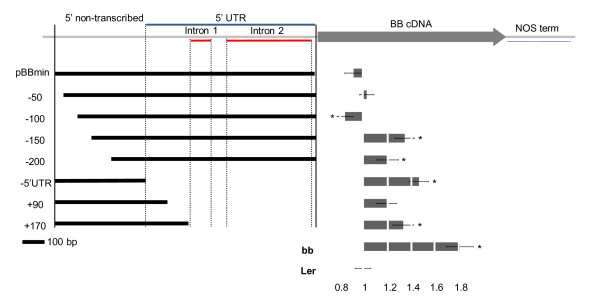
**Promoter deletion analysis**. The initial construct of pBBmin is shown on top. The gray arrow indicates *BB *cDNA; while 5' UTR and introns are marked with blue and red lines, respectively. Lines below indicate the promoter constructs for the sequential deletion series. Bars next to the constructs are the means of the petal measurements. Error bars are SE and significant changes compared to L.*er *are marked with * (*p *< 0.05). All measurements are significantly different from *bb-1 *(*p *< 0.03)

A 3.5 kb genomic fragment of the *BB *locus consisting of 1.3 kb of 5' non-transcribed promoter sequence, 669 bp of 5' UTR containing two introns, the coding sequence (CDS) and 199 bp of 3' UTR contains all regulatory elements sufficient for rescuing levels of *BB *expression [6]. In order to isolate *cis*-elements required for the regulation of *BB *expression, we fused 1035 bp upstream of the *BB *start codon to a *BB *cDNA. This initial construct, termed minimal promoter (pBBmin), consists of 366 bp non-transcribed sequence and the entire 5' UTR and is sufficient to rescue the petal overgrowth phenotype of *bb-1 *mutant plants, indicating that it contains all necessary regulatory elements for *BB *expression regulation (Figure 1).

To further delimit regulatory elements within this stretch of sequence we removed successive 50-bp fragments from the 5' end of the non-transcribed sequence in four steps, until only 141 bp upstream of the transcription initiation site remained. In a parallel experiment only the 366 bp of non-transcribed sequence were fused to an alternative 5' UTR (omega sequence, [10]) or to 90 bp or 170 bp of the endogenous 5'UTR sequence. The resulting seven promoter deletion constructs (illustrated in Figure 1) were all fused to the *BB *cDNA and transformed into *bb-1 *mutant plants. We generated lines homozygous for a single transgene insertion derived from three independent primary transformants and measured petal size of 3-5 plants as a read-out for transcriptional activity of the promoters to be tested. The results from the individual lines for each construct are shown in Additional file 1: Figure S1. This assay allowed us to determine not only the qualitative functionality of the promoter sequences, i.e. the correct spatial expression pattern in petals, but also quantitatively assess the levels of expression. This is because petal size strictly depends on *BB *dosage, with a mere two-fold reduction in expression in heterozygous plants leading to a significant increase in petal size [6]. We note that this approach might miss specific regulatory sequences that are only active in organs other than petals. For better visualisation we normalised the average petal sizes to the Landsberg *errecta *(L.*er*) wild-type value. To test for significant changes we performed a pair-wise *t*-test on all plants derived from one construct compared to wild-type and to account for multiple testing we used the Benjamini-Hochberg correction [11]. While removing the first 50 bp from the 5’ non-transcribed sequence did not affect the rescuing activity of the construct, eliminating a further 50 bp resulted in one transgenic line in smaller than wild-type petals, suggesting the existence of a potential negative regulatory element within this second 50-bp fragment. However, as two of the three lines measured did not show any significant changes to wild type, this result needs to be confirmed independently. Removal of 150 bp from the 5' non-transcribed promoter sequence resulted in an almost 40% increase in petal size in the transgenic plants (compared to an 80% increase vs. L.*er *in non-transgenic *bb-1 *mutants), indicating that the third 50-bp fragment contains important positively acting promoter elements. An effect of similar strength was observed, when parts or all of the 5' UTR were removed (constructs "-5'UTR" and "+170", Figure 1), indicating that the 5' UTR also contains sequences important for normal *BB *expression and that the non-transcribed sequence on its own is not sufficient to condition rescuing levels of *BB *expression. As removal of sequences from the 5' UTR or from the non-transcribed region only caused a loss of rescuing activity to what is seen in *bb1/+ *heterozygous plants, there appear to be two independent regulatory inputs, acting on either of these regions, with only their combined action leading to wild-type *BB *expression levels. Also, positive and negative inputs seem to be integrated by the *BB *promoter.

As *cis*-regulatory elements within the 5' UTR could affect *BB *transcription as well as its translation, which we cannot monitor at present, we decided to focus our further analysis on the non-transcribed sequences.

### The *BB *CDS is strongly conserved within Brassicaceae

Putative E3 ubiqutin ligases related to BB can be found in several plant species [6], suggesting that *BB *function is conserved among plants. This would open up the possibility to use phylogenetic footprinting (see below) to identify *cis-*regulatory promoter elements by virtue of their conservation. As a step towards isolating the non-transcribed promoter sequences, we first isolated the *BB *genomic coding sequence (CDS) from seven species in six different Brassicaceae genera: *Arabidopsis thaliana, Arabidopsis lyrata, Arabis alpina, Cardamine hirsuta, Iberis amara, Sisymbrium officenale *and *Thalspi perfoliata*. Together these genera represent examples for almost the entire Brassicaceae family [12,13].

**Figure 2:**
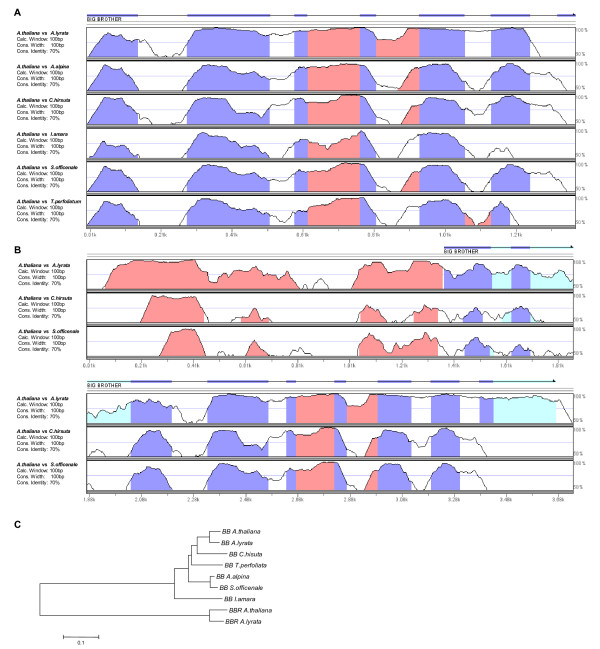
**VISTA plot of *BB *CDSs**. **a**) VISTA plot of pair-wise comparisons of different *Brassicacea *species to *A. thaliana*. Intron and exon annotation of *A. thaliana *is shown on top (exons are represented by thick lines, introns by thin lines) and filled portions of the graphs indicate conservation of more than 70% with a width of at least 100 bp (red for intron, blue for exon sequence). **b**) VISTA plot of *BB *CDS and promoter sequences *of A. lyrata, C. hirsuta *and *S. officinale*. Note high conservation within non-transcribed region (color code same as in **a**, light blue for 5' UTR). **c**) Phlyogenetic tree of *BB *CDSs using *BBR *of *A. thalina *and *A. lyrata *as outgroup.

The genomic *BB *sequences for *A. thaliana *and *A. lyrata *were isolated from public databases (see Materials and Methods). For the other species, we used primers designed against the *BB *CDS from *A. thaliana *and amplified the CDS sequences by PCR. All sequences were compared to the *A. thaliana *reference using the mVISTA tool [14,15]. The alignment of the isolated *BB *CDS showed a very high degree of conservation within all isolated genera (Figure 2a). The most divergent sequence stretches were found in regions corresponding to introns within *A. thaliana *(marked in white on the VISTA plot in Figure 2a), suggesting that the intron-exon structure of *BB *is well conserved. Interestingly, not all introns show such a low conservation. In the VISTA plot using a 100-bp sliding window analysis with a minimum conservation width of 100 bp, introns in the middle part of the BB CDS show levels of conservation above 70% throughout the seven species (marked in red on the VISTA plot in Figure 2a), suggesting that these introns contain functional elements. To confirm that the isolated sequences are really the CDS of the orthologues of *BB *and not of the homologous *BIG BROTHER-RELATED *(*BBR*, At3g19910) gene, we performed a phylogenetic analysis using the CDS of the *A. thaliana *and *A. lyrata BBR *as outgroup. In this analysis all *BB *CDS clearly clustered together, indicating that we isolated the orthologues of *BB *and not of *BBR *(Figure 2c).

For four of the seven species (*A. thaliana*, *A. lyrata*, *C. hirsuta* and *S. officinale*) we were able to isolate larger genomic fragments containing also 5’ non-transcribed sequences. The VISTA alignment against *A. thaliana *using these larger fragments showed a high level of conservation also within the 5' UTR and the approximately 300 bp of non-transcribed sequence (Figure 2b). Taken together, these results show that the *BB *gene is well conserved within Brassicaceae. The conservation is not only limited to the transcribed exon sequence of BB, but also non-transcribed and intronic regions show relatively high levels of sequence similarity.

### Phylogenetic footprinting reveals strong conservation of the 5' non-transcribed sequence

In addition to the protein coding sequence, regulatory elements important for proper qualitative and quantitative expression also tend to be conserved to maintain the function of a given genomic locus. The high sequence conservation of *BB *CDSs and non-transcribed sequences within Brassicaceae encouraged us to use phylogenetic footprinting, i.e. to systematically compare the non-transcribed regions of the *BB *loci to further delimit *cis*-regulatory elements based on their conservation across taxa [7-9]. To this end, we used thermal asymmetric interlaced-PCR (TAIL PCR) to amplify genomic sequences extending 5' from the highly conserved *BB *CDS [16,17]. We were able to isolate approximately 1 kb of non-transcribed sequence from *T. perfoliatum, A. alpina, S. officinale, I. amara *and *C. hirsuta*. For *A. lyrata *and *Brassica oleracea *we found aligning sequences in available databases (see Materials and Methods). All isolated DNA sequences contained the entire minimal promoter which we determined in previous experiments (Figure 1).

**Figure 3:**
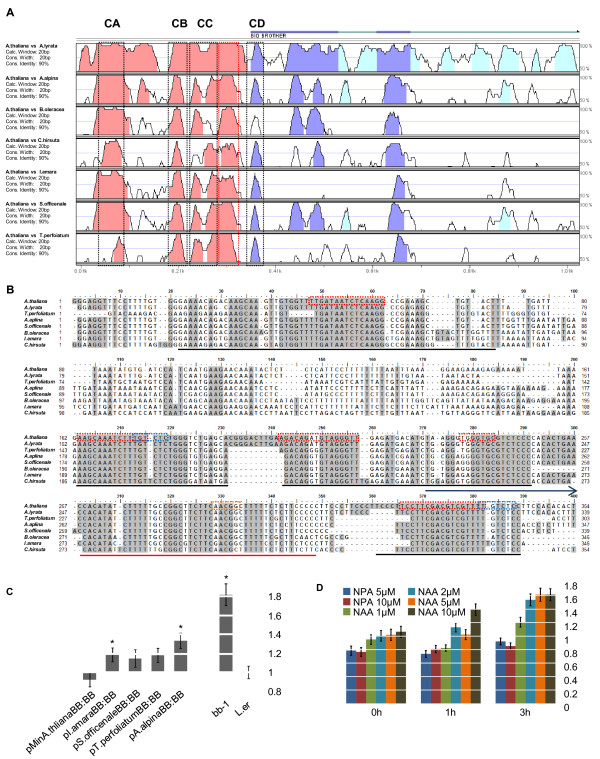
**Phyogenetic footprinting of *BB *promoter**. **a**) VISTA plot of pair-wise comparisons of different Brassicaceae and *A. thaliana*. Graph illustrates a 20 bp sliding window comparison and conservation of more than 90% with a minimum width of 20 bp is marked colour (dark blue for exon in 5' UTR, light blue for intron in 5' UTR, red for non-transcribed sequence). Blocks of high conservation are indicated as boxes, black elements were analysed further and the red box was not analyzed as no binding motif was predicted by FootPrinter. **b**) Sequence alignement of the non-transcribed BB promoter. Highly conserved blocks are underlined. Motifs predicted by FootPrinter are indicated as red boxes. ARF binding sites are shown as blue boxes and MYB binding site as orange box. The blue arrow above positions 370 to 400 indicates 5' UTR sequence. **c**) *bb-1 *rescue experiment using different Brassicaceae promoters normalised with L.*er*. Error bars indicate SE and significant changes to L.*er *are marked with * (*p *< 0.05). All measurements are significantly different to *bb-1 *(*p *< 0.03). **d**) Auxin induction assay measuring LUC expression after 1h (T1) and 3h (T2). All measurements were normalized to the values for the H_2_O control at the respective time points, error bars are SE.

The alignment of all *BB *promoter sequences showed a very high degree of conservation within the non-transcribed sequence. 174 bp of the 366 bp of the non-transcribed sequence (47.5%) of *A. thaliana *showed conservation of up to 100% within all Brassicaceae promoters analysed. In comparison only 24 bp of the 669 bp of 5' UTR (3.5%) showed conservation to such high levels (Figure 3b). This indicates that high levels of sequence diversity can be found between the *BB *loci in the selected species, and that conversely the high levels of conservation within the non-transcribed sequence are not only due to the close phylogenetic relationship of the taxa in question.

Pair-wise alignments of the individual sequences with the *A. thaliana *sequence using mVISTA [14,15] showed that the highly conserved regions fall into five blocks of 24-53 bp according to the *A. thaliana *promoter sequence. For better visualisation of the high similarity we used a 20 bp sliding window analysis in which blocks with 90% similarity or more are marked in color (solid red or blue graphs in Figure 3a). The first four blocks were located within the non-transcribed region and only one block of 24 bp was located within the 5' UTR right next to the annotated transcriptional start site (see boxes in Figure 3a and underlined stretches in Figure 3b). Three of the four conserved blocks within the non-transcribed sequence were located in the proximal 165 bp upstream from the transcription start site (Figure 3a, b), in the sequence that was still present in the "-200" construct tested in Figure 1.

To identify putative regulatory motifs within this alignment, we scanned for putative *cis*-regulatory elements using the FootPrinter algorithm [18]. This algorithm uses a motif discovery approach on a comparison of a set of homologous sequences. The program searches for defined *k-*mers (motives), one from each sequence, within the set of sequences allowing for nucleotide substitutions within the *k-*mers depending on the phylogenetic relation, i.e. more closely related species are expected to share more similarity. In this way the algorithm allows the identification of regulatory *cis*-elements, even if promoter sequences are too divergent to be accurately aligned [18] like the 5' UTR of the *BB *minimal promoter sequence. In our alignment the FootPrinter software predicted four putative binding sites within the non-transcribed sequence and one at the start of the 5' UTR (see red dashed boxes in Figure 3b), all of which were within the highly conserved sequences. However, the software did not predict a binding site within all conserved sequences. Two of the putative binding sites were located within one block of conserved sequence whereas one other block did not contain any predicted binding site (see Figure 3a red block and red underlined sequence in Figure 3b).

Taken together the phylogenetic footprinting of *BB *promoters from eight Brassicaceae species showed that the non-transcribed sequences and the area around the transcriptional start site are highly conserved, suggesting the presence of *cis*-regulatory elements. The *cis*-elements predicted to be present in the 5' UTR based on our previous results (Figure 1) were not found, suggesting that the input pathways controlling *BB *expression via *cis*-elements within this region may have diverged. As one of the highly conserved blocks was located right in front of the annotated transcriptional start site and did not contain any predicted *cis*-elements according to the FootPrinter results, this region may contain mainly sequence elements important for basic transcription initiation. Therefore we focused our further characterization on the four other highly conserved blocks, which we termed conserved block A, B, C and D (CA to CD, Figure 3a black boxes).

### Putative auxin binding sites are not functional

To test whether the conserved domains of the pBBmin promoter contain any known binding sites for *trans*-acting transcription factors we searched for binding sites within the 5 conserved blocks using the PLACE database [19,20]. PLACE predicted several putative binding sites for transcription factors. However, most of the binding sites showed matches only in 4 nucleotides and were therefore ignored. Interestingly, block CB and CD contained full matches of the AUXIN REPONSE FACTOR (ARF) binding site (TGTCTC, marked as blue boxes in Figure 3b). Another putative binding site for a MYB transcription factor was predicted within the conserved block upstream of the transcriptional initiation site.

In order to test whether the predicted ARF binding sites are functional we performed an auxin induction assay in seedlings on a pBBmin construct fused to the *LUCIFERASE *(*LUC*) reporter gene. LUC activity was measured after 1 h and 3 h induction with different concentrations of the auxin transport inhibitor NPA or the auxin analogon NAA and measurements normalized against the mock control. For the treatments with NPA we were not able to find significant changes and NAA only lead to a modest induction (Figure 3d). For comparison, Pufky et al. [21] showed that genome wide a large number of genes containing the ARF binding site are upregulated more than two-fold after 60min of induction. Also, Ulmasov et al. [22] showed an 8-fold induction by auxin of GUS fused to a promoter containing the TGTCTC motif. These results are in line with previous published literature showing that the genomic protein fusion of BB to GUS could not be induced by auxin. Also, the *bb-1* mutant was shown to have no altered sensitivity to any tested phytohormones, suggesting that BB acts independently of the major phytohormones [6]. Therefore we concluded that the predicted ARF binding sites are most likely not functional.

### Brassicaceae promoters are functional in *A. Thaliana*

One important assumption in the phylogenetic footprinting analysis is that conservation of sequence also represents functional conservation. However, as the sequences around the highly conserved elements in the different Brassicaceae species show a high level of diversity, it remains to be tested whether the mere conservation of small sequence stretches results in functional conservation of the promoters. To address this, we tested the functionality of some of the isolated *BB *promoters from other species in *A. thaliana*. To this end, the *BB *promoter sequences of *A. alpina, I. amara, S. officinale *and *T. perfoliatum *were fused to the *BB *cDNA from *A. thaliana *and transformed into *bb-1 *mutant *A. thaliana *plants. All chosen promoter sequences showed high levels of conservation within the non-transcribed sequence; the 5' UTR, however, was very variable (Figure 3a).

For all constructs, we again established homozygous lines derived from three independent primary transformants each and measured their petal sizes. All tested lines showed a significant decrease in petal size, suggesting that the respective promoters are largely functional in *A. thaliana *(Figure 3c). The level of phenotypic rescue did not depend on the similarity within the 5' UTR, as also the promoters of *I. amara *and *T. perfoliatum *with their divergent 5' UTR sequences relative to *A. thaliana *were able to rescue the *bb-1 *phenotype to a similar extent as the other promoters (Figure 3c). These results suggest that the sequence conservation in the 5' non-transcribed region is indeed indicative of functional conservation. Whether in the *I. amara *and *T. perfoliatum *promoters other sequences fulfill the same function as the 5' UTR sequences in *A. thaliana *will need to be resolved by further experimentation.

### Conserved *cis*-elements are important for regulation of *BB *expression

**Figure 4:**
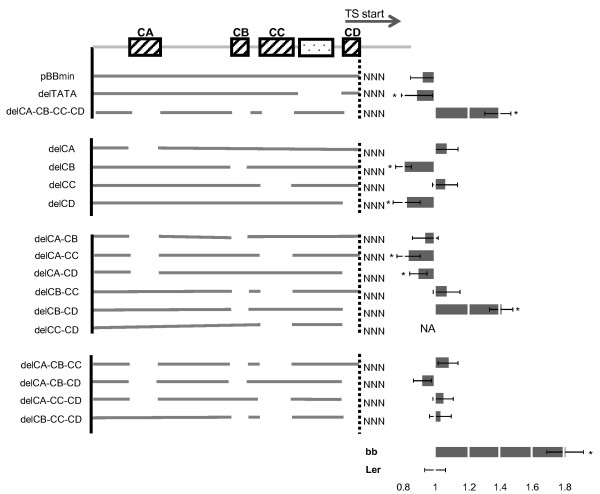
**Promoter deletion assay for conserved elements**. Top line indicates the non-transcribed sequence of pBBmin with highly conserved blocks shown as blocks (dashed boxes contain predicted binding motifs from FootPrinter, dotted box does not contain a predicted binding motif). Deletion constructs are illustrated in lines on the left side. Bars on the right side indicate mean of petal measurements. Significant changes relative to L.*er *are marked with * (*p *< 0.05). All measurements are significantly different to *bb-1 *(*p *< 0.03)

To determine the function of the predicted conserved *cis*-elements in *A. thaliana*, we implemented a more refined promoter deletion analysis. In an initial experiment we removed all conserved elements together (delCA-CB-CC-CD in Figure 4). As *BB *expression in *A. thaliana *is also influenced by *cis*-elements within the 5' UTR we predicted that such a construct would not completely abolish expression from this promoter, but should significantly decrease the rescuing activity, if the deleted elements play an important role. Similarly, we also addressed the function of each conserved element individually. To this end we removed each conserved block independently from the minimal promoter. To control for the influence of transcription initiation site we also included a single deletion of the 65 bp neighbouring the transcription start site. All constructs were fused to the *BB *cDNA from *A. thaliana *and transformed into *bb-1 *mutants. As before, we assayed the activity of the promoter constructs by analysing homozygous transgenic lines derived from three individual primary transformants.

The results of this deletion series are summarized in Figure 4. As before, the average petal size is normalized to the L.*er *wild-type value. The deletion of all four conserved elements led to a significant decrease of the rescuing activity, resulting in a 40% increase of petal size relative to wild-type and control transgenic plants with the unmodified minimal promoter construct. Thus, the removal of the four highly conserved blocks of promoter sequence strongly impairs functionality (Figure 4). By contrast, removal of none of the elements by themselves caused a decrease of the rescuing activity. Rather, weak repressive effects of CB and CD are suggested by the slightly smaller than wild-type petals in the corresponding transgenic lines. This suggests that more complex interactions between the CA, CB, CC and CD regions may be involved in *BB *expression regulation.

To address such potential interactions between different binding sites and thus potentially different inputs, we generated deletions of pairs of the four conserved elements and measured their functionality as before. The analysis of the different double deletions showed a complex picture. Of particular interest was the result of deleting both CB and CD. While both single deletions seemed to enhance the activity of the promoter derivatives (see above), combined removal of CB and CD interfered with the rescuing activity of the construct to a similar level as removing all four conserved elements did.

As a next step, we deleted all possible combinations of three of the four conserved elements, resulting in constructs with only one of the elements remaining. To our surprise, measurements of petal sizes in the resulting transgenic lines indicated that all of the deleted constructs were still able to rescue the *bb-1 *phenotype to essentially wild-type levels. Thus, with respect to the combination of CB and CD, the additional removal of either CA or CC restored the activity of the promoter. This suggests that in the absence of the CB and CD sequences, repressive factors bind to the modified promoter, and that this binding is abolished when either CA or CC are removed in addition.

Taken together, the functional analysis of the conserved elements within the non-transcribed sequence suggests that these elements harbor binding sites for important *trans*-acting factors. However, none of the analyzed elements seems to be essential by itself (i.e. they can all be individually deleted without a loss of promoter activity), suggesting that multiple, potentially interacting pathways promote *BB *expression.

## Conclusions

In summary, this study has identified phylogenetically strongly conserved sequences within the *BB *non-transcribed promoter region that are likely to function as important *cis*-regulatory elements. It also highlights the complexities involved in precise quantitative regulation of gene expression that is likely to involve and integrate several input pathways acting independently or in a combinatorial manner. A future analysis of the *trans*-acting factors that bind to the identified elements will be required to understand how the level of *BB *expression is determined at the molecular level to ensure organ growth up to the appropriate size.

## Methods

### Plant material and growth

L.*er *wild-type was used as reference for full functionality of the tested constructs. As negative control we used *bb-1 *plants [6]. Seeds from *Arabis alpina, Iberis amara, Sisymbrium officinale *and *Thlaspi perfoliatum *were kindly provided by the Botanical Gardens of the Universities of Hohenheim, Tübingen and Würzburg. Plants were grown on soil under standard conditions in 16-h-light/8-h-dark cycle.

For petal measurements plants were grown under the same light conditions with an additional temperature cycle of 21°C during light and 16°C during dark. Of each plant petals of 2 flowers were harvested on sticky tape. After scanning the petals using a 3600 dpi scanner, petal size was determined of four petals per plant using the ImageJ software.

### Cloning of constructs

For the sequential deletion series promoter fragments were amplified using PCR introducing *Hin*dIII and *Bam*HI sites at the 5' and 3' ends, respectively. Deletions of the conserved elements were introduced using a double PCR strategy. 3' and 5' ends of the deletion were amplified independently and fused in a second PCR step introducing again *Hin*dIII and *Bam*HI sites at the 3' and 5' end, respectively. For multiple deletions this procedure was repeated using single, double or triple deletion constructs as templates. Brassicaceae promoters were amplified using specific promoter primers introducing a 5' *Hin*dIII and 3' *Bam*HI site. All primer sequences used are provided in Additional file 2: Table S1. By making use of the introduced *Hin*dIII and *Bam*HI sites all amplicons were subcloned into a binary pBarMAP vector containing a *BB *cDNA and terminator sequences preceded by unique *Hin*dIII and *Bam*HI sites. All constructs were transformed into *bb-1 Arabidopsis *plants using the floral dip method.

### Isolation of BB genomic coding sequences

*BB *genomic coding sequences from *A. alpina, I. amara, S. officinale *and *T. perfoliatum *were isolated from genomic DNA using different combinations of *A. thaliana *specific primers binding at the 5' and 3' end (HBo095 to HBo098, Additional file 2: Table S1), respectively. Amplicons were sequenced and aligned to *A. thaliana *sequence isolated from the TAIR database. The *BB *genomic coding sequence of *C. hirsuta *was amplified using the same primers; however, instead of genomic DNA a corresponding P1 BAC was kindly provided as template by Miltos Tsiantis. *A. lyrata *sequences were isolated in the sequence trace archive of NCBI [23] using a BLAST search with *A. thaliana *sequence.

### Isolation of *BB *promoter sequences

*BB *promoter sequences of *A. thaliana *were isolated from the TAIR [24] database collection. For *A. lyrata *and *B. oleracea *the *A. thaliana *sequence was used in a BLAST search of the NCBI trace archive or the Arabidopsis Thaliana Integrated Database [25], to isolate corresponding *BB *promoter sequences.

Promoter sequences of *A. alpina, I. amara, S. officinale *and *T. perfoliatum *were isolated following the Thermal Asymmetric Interlaced PCR (TAIL) protocol as published online [26]. As gene specific primers we used HBo124, HBo123 and HBo79 (Additional file 2: Table S1). The isolated amplicons were subcloned into pGEM-T (Promega) vector and sequenced. Matching sequences were determined by performing a BLAST search against the TAIR *A. thaliana *database.

For *C. hirsuta *promoter sequences were directly amplified from the provided P1 BAC using HBo72 and HBo124 primers.

### Computational and statistical analysis

VISTA alignments of the isolated promoter sequences were done using the online available VISTA tools [27] The Footprinter 3 analysis was done using the online resources of the University of Washington [28]. The phyogenetic tree analysis of BB genomic coding sequences was conducted using the MEGA version 5 software package [29].

Statistical evaluation was performed using a two-tailed pair-wise *t*-test of all measured plants of one construct, about five plants for each of three independently derived homozygous transgenic lines, i.e. approx. 15 plants in total. Petal sizes were compared to measurements of an equal number of L.*er *wild type plants. Benjamini-Hochberg correction was used to account for multiple testing [11].

### Hormone induction

Hormone induction assays were performed using a *pBBmin:LUC *fusion. In this construct the *BB *cDNA was replaced by a cDNA of the *LUCIFERASE *gene using appropriate restriction sites. The construct was transformed into L.*er *wild type background and strong *LUC *expressing lines were selected. For the assay, individual homozygous *pBB:LUC *plants were germinated on plates. Seedlings were then sprayed with 5 μM and 10 μM NPA and 1 μM, 2 μM, 5 μM and 10 μM NAA or H_2_O as negative control. *LUC *expression was measured using a NightOwl (Berthold) ccd camera before induction (T0), after 1 h (T1) and after 3 h (T2). Measurements of approx. 60 seedlings were averaged and normalised with the averaged of the water control of the respective time point.

## Authors' contributions

HB participated in the design of the study, carried out all the construction of the used material, carried out all the experimental work, and participated in writing the manuscript. ML conceived the study, participated in its design and coordination and participated in writing the manuscript. All authors read and approved the final manuscript.

## Supplementary Material

Additional file 1 **Figure S1**. Relative petal size of independent transformant lines. Click here for file

Additional file 2 **Table S1**. Table of oligonucleotides used. Click here for file
