# Supplementary material for: Expression of the central growth regulator BIG BROTHER is regulated by multiple cis-elements
Source: BMC Plant Biol. 2012 Mar 20;12:41. doi: 10.1186/1471-2229-12-41 (PMC3362746; doi:10.1186/1471-2229-12-41)
Supplement: Additional file 2 — Table S1. Table of oligonucleotides used. [file 1471-2229-12-41-S2.PDF]

Table S1: List of oligonucleotides used

Name	Length	Oligo Sequence	Description
HBo014 (Oligo18)	25	ATAGGATCCAGCTACTGCAATCGAG	
HBo015 (For-1040-HindIII)	22	AAACAAAGCTTGTCTGCAGTAG	Amplification of Pmin (pos. -1040) BB HindIII added
HBo016 (For-990-HindIII)	24	GTAAGCTTTTGTGGGAAAACAGAC	Amplification of Pmin-50 HindIII added
HBo017 (For-940-HindIII)	28	CGAAAGCTTTACTTTTGATTTAAATATG	Amplification of Pmin-100 HindIII added
HBo018 (For-880-HindIII)	26	CTCAAGCTTTTTTTTTAATTTAAAGG	Amplification of Pmin-150 HindIII added
HBo019 (For-820-HindIII)	23	CTAAGCTTGGGACTTGAAGACAG	Amplification of Pmin-200 HindIII added
HBo020 (Rev-690-BamHI)	22	ACGGGATCCAAGGAAGGGAAGG	Amplification of minProm (TS-start) BB BamHI added
HBo021 (Rev-600-BamHI)	20	GGGATCCGGAGGAGCAGAGG	Amplification of Pmin+90
HBo070 (Rev-510-II-BamHI)	25	TACGGATCCTTAGTGGAAGGAGGAG	Amplification of Pmin+170 new (uTAA maintained)
HBo024 (DelTATArev)	31	AAACGACGTCGTGGTTCAGTGTGGGGAGACG	amplification of delTATA rev
HBo025 (DelTATAfor)	33	ACACTGAACCACGACGTCGTTTTGTCTCCTTCC	amplification of DelTATA for
HBo028 (Oligo16)	25	ATAAGCTTAAGAAGAAGACGGAGAA	
HBo095 (BBgenfor1)	27	ATGGAGATAATAGACCAGTGGAAGATG	amplification of genomic BB including ATG
HBo096 (BBgenrev1)	23	CAAAGACCTCGCTGTTACACACC	amplification of genomic BB binds in last exon
HBo097 (BBgenfor2)	21	GGTGAACGTTGCTGATGATCC	amplification of genomic BB. Primer binds in second exon
HBo098 (BBgenrev2)	25	CATTCAGAATGATACACATGCTTGC	amplification of genomic BB. Primer binds in second last exon
HBo123 (BB-A85consRev)	22	AGGCCCCTGAGCCGCACCACCA	primes reverse in conserved streach of BB at pos. 85, used for TAIL
HBo124 (BB-A280consRev)	24	TTCATGGTCCAGTACAGATTGTCC	primes rev in conserved streach of BB at pos280, used for TAIL
HBo079 (BBprom-rev2)	23	TCCAGTAGCAGCATAAGGGAAAC	Primer close to BB ATG to amplify BB promoter, used for TAIL
HBo072 (BBprom-for)	21	GCCTTGTCGGACGTGGAGCTG	use to amplify BB promoter for footprinting
HBo117 (AD1)	16	NGTCGASWGANAWGAA	Degenerated primer for TAIL PCR
HBo118 (AD2)	16	TGWGNAGSANCASAGA	Degenerated primer for TAIL PCR
HBo119 (AD3)	16	AGWGNAGWANCAWAGG	Degenerated primer for TAIL PCR
HBo120 (AD4)	16	STTGNTASTNCTNTGC	Degenerated primer for TAIL PCR
HBo121 (AD5)	15	NTCGASTWTSGWGTT	Degenerated primer for TAIL PCR
HBo122 (AD6)	16	WGTGNAGWANCANAGA	Degenerated primer for TAIL PCR
HBo133 (Iberis-pBB-HindIII-for)	32	AAGCTTCAATTTACCCTACAACTGAGTAGACG	cloning primer for pBB of Iberis.
HBo134 (Iberis-pBB-BamHI-rev)	30	GGATCCAGCTACTACAACTAAAGTTGATAC	cloning primer for pBB of Iberis.
HBo135 (Sisy-pBB-HindIII-for)	32	AAGCTTACATCTATCTCTCTCTGTGACAATAC	cloning primer for pBB of Sisymbrium officenale.
HBo136 (Sisy-pBB-BamHI-rev)	29	GGATCCTTGAGCTGCTGGTATTGAGTAAG	cloning primer for pBB of Sisymbrium officenale.
HBo137 (Arabis-pBB-HindIII-for)	30	AAGCTTCCTTTTGTGGGAAAACAGACAAGC	cloning primer for pBB of Arabis alpina.
HBo138 (Arabis-pBB-BamHI-rev)	31	GGATCCAGTGTTGTTGGGGTTACTAGGAGAG	cloning primer for pBB of Arabis alpina.
HBo139 (Thlaspi-pBB-Hind-for)	29	AAGCTTCGCCATGTACAAAGACGAAGAAG	cloning primer for pBB of Thlaspi.
HBo140 (Thlaspi-pBB-BamHI-rev)	30	GGATCCGATGAAGAGTTAGAAGGTAACTCC	cloning primer for pBB of Thlaspi

HBo141 (delta-CAfor)	38	AGGTTTCCTTTTAAAGCTGTACTTTTGATTTAAATATG	cloning primer forward for CA element
HBo142 (delta-CArev)	34	AAGTACAGCTTTAAAAGGAAACCTCCCTTCCATG	cloning primer revese for CA element
HBo143 (delta-CBfor)	36	GAAAGAGAAAAATTCGGGACTTGAAGACAGATGTAG	cloning primer forward for CB element
HBo144 (delta-CBrev)	37	TCTTCAAGTCCCGAATTTTTCTCTTTCTTCCTTTAAA	cloning primer reverse for CB element
HBo145 (delta-CCfor)	36	CGGGACTTGAAAACCACATATCTTTTTGCCGGCTTC	cloning primer forward for CC element
HBo146 (delta-CCrev)	35	AAAGATATGTGGTTTTCAAGTCCCGTGCTCAGACC	cloning primer reverse for CC element
HBo147 (delta-CCrev2)	35	AAAGATATGTGGTTTTCAAGTCCCGAATTTTTCTC	cloning primer reverse for delta CC on pBBmin CB for CB-CD deletion
HBo148 (delta-CDfor)	34	CTTCCCTTCCCTTCCACACACTCTTTCCTCTCTC	cloning primer forward for delta CD element
HBo149 (delta-CDrev)	30	GAGTGTGTGGAAGGGAAGGGAAGGGAAGGG	cloning primer reverse for delta CD element
