# Supplementary material for: Expression of the central growth regulator BIG BROTHER is regulated by multiple cis-elements
Source: BMC Plant Biol. 2012 Mar 20;12:41. doi: 10.1186/1471-2229-12-41 (PMC3362746; doi:10.1186/1471-2229-12-41)
Supplement: Additional file 1 — Figure S1. Relative petal size of independent transformant lines. [file 1471-2229-12-41-S1.PDF]

Figure S1: Relative Petal Size of Independent Transformant Lines

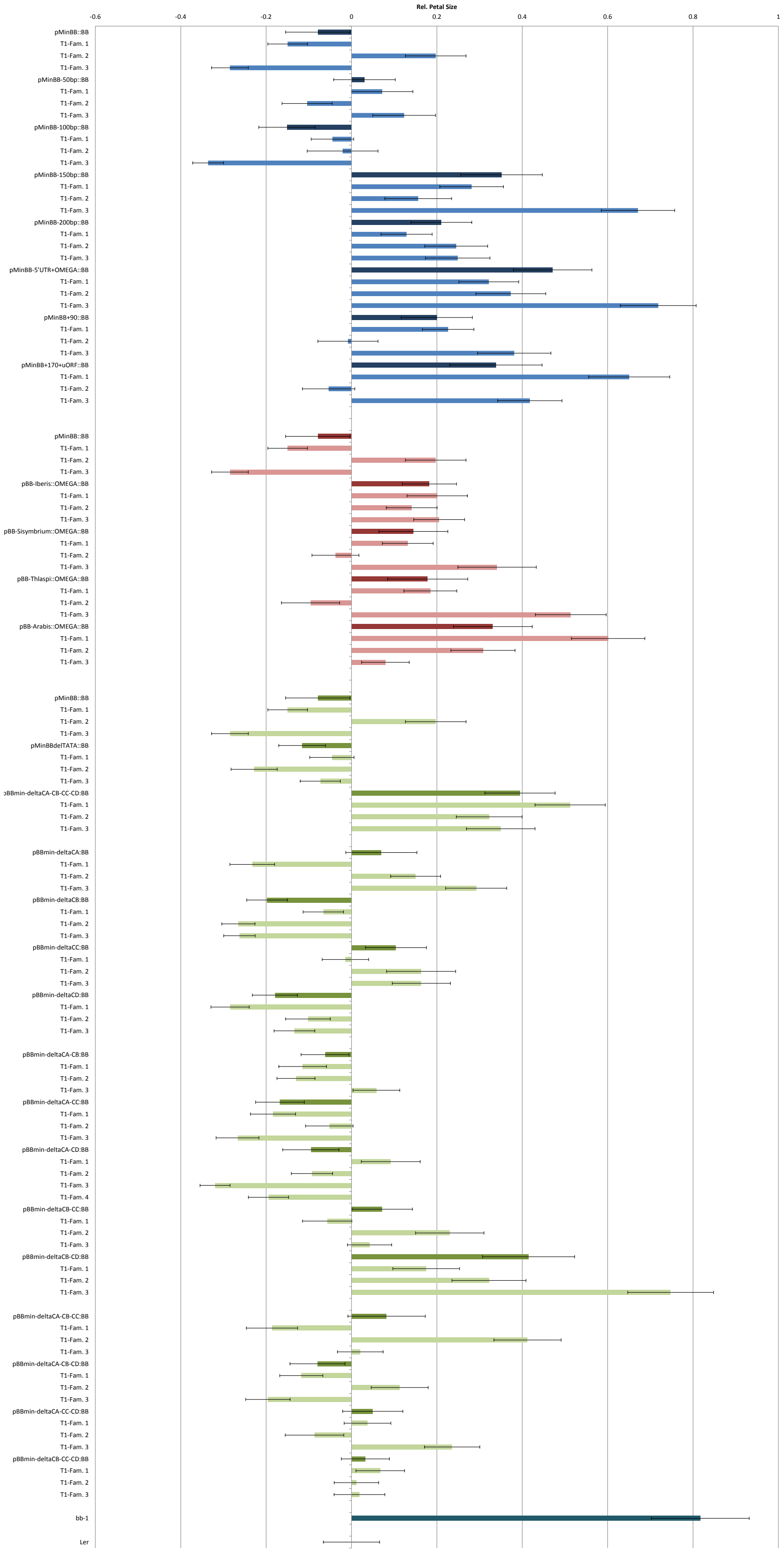

Figure S1: Petal size measurements of individual transformant lines. All measurements were normalized to L*er* WT and error bars indicate SE. Blue bars represent measurements of constructs in Figure 1, red in Figure 3 and green in Figure 4. Dark colored bars are the mean of all lines per construct as shown in the main figures and light colors indicate means of the independent transformant lines.
